# The Occurrence and Significance of B. Welchii in Certain Wounds

**Published:** 1919

**Authors:** 


***The Occurrence and Significance of B. Welchii in Certain Wounds.*** By Capt. J. L. Stoddard M. R. C., U. S. Army. *Journal of the American Medical Association*, October 26, 1918.

The author gives a short review of the studies made up to the present time on B. Welchii, and records his own observations, which may be summarized as follows :

*The Occurrence of B. Welchii in Wounds after Thorough C. C. S. Treatment.* In a consecutive series of 137 wounds, aged from 1 to 11 days, in patients arriving at a base hospital after C. C. S. treatment, B. Welchii was obtained by culture in 23 o/o. Cases with more than extremely few B. W. organisms made 14 o/o of series, distributed as follows :

| | |
|---|---|
| (1) One-day wounds. . . . . . . . . . . . . . . | 18 o/o |
| (2) Wounds of 2 days. . . . . . . . . . . . . . | 18.6 o/o |
| (3) " " 3 " . . . . . . . . . . . . . | 8 o/o |
| (4) " from 4 to 7 days . . . . . . . . . . | 11 o/o |

The influence of excision at first seemed nil, for according to field medical cards B. Welchii was present in 23.5 o/o of the excised cases, and in 19.4 o/o of the non-excised ones; this was due to the fact that the wounds excised were those in which B. Welchii was most likely to occur.

*Incidence of Gas-infection.* Definite muscle gas-infection occurred in 2.9 o/o of the series. Including slightly doubtful cases, the incidence was 5.1 o/o. Of the cases with B. Welchii on culture, those with unquestionable infection numbered 13 o/o, and including the slightly doubtful cases 22.5 o/o.

*The Persistence of B. Welchii in the Wounds.* For further studies, cases of gas-infection were included from a previous non-consecutive series. These cases were practically all in excised wounds. It was found that the cultures of B. Welchii tend to become negative by the 8th day when no gas-infection has occurred, but to last until the 12th day or later in cases with infection. Even reckoning the day of operation as the starting point, persistence is distinctly greater in gas-infected wounds. In cases without gas-infection, the numbers of bacilli tend to decrease early in the history of the wound, while in cases with gas-infection there is a definite tendency to persist in greater numbers until the 8th day and later. Persistence in cases without gas infection is probably due to previously unrecognized local infection or to small anerobic foci in large wounds in which excision is not practicable; persistence after gas-infection may be explained by a gradual riddance of the bacilli from the invaded but not altered tissues.

*The Value of Examination for B. Welchii.* The presence of large numbers of B. Welchii in the wound is good evidence of an active gas-infection. Moderate numbers cannot lead to any conclusion, and the absence of the bacilli is no final proof of the absence of infection, for negative cultures are occasionally obtained when B. Welchii is not a factor in the infection, or when the infection begins deep about a foreign body. These facts regarding the value of smear and culture, with reference to the diagnosis of gas-infection in wounds, appear to rest on the inability of B. Welchii to grow for a long period on surgically clean wounds, thus preventing the occurrence of large numbers when no infection is present, and on the occurrence of large numbers of B. Welchii in the exuded serum of the infected cases, thus causing the highly positive smears.

*Relation to Suture.* As far as the evidence of a small number of cases goes, the presence of B. Welchii is not a contra-indication for suture. B. Welchii may persist harmlessly as long as 5 days after suture.

